# The Role of Neuronal Pentraxin 2 (NP2) in Regulating Glutamatergic Signaling and Neuropathology

**DOI:** 10.3389/fncel.2019.00575

**Published:** 2020-01-08

**Authors:** Georgina Chapman, Ushananthini Shanmugalingam, Patrice D. Smith

**Affiliations:** Department of Neuroscience, Carleton University, Ottawa, ON, Canada

**Keywords:** AMPA receptor, Narp, neuronal pentraxins, glutamatergic neurotransmission, excitotoxicity, neuropathology

## Abstract

Pentraxins are a superfamily of evolutionarily conserved proteins that are characterized by their multimeric architecture and their calcium-dependent binding. They can be broadly grouped into two subfamilies: short pentraxins and long pentraxins. Pentraxins regulate many processes in the brain as well as the periphery. Neuronal pentraxin 2 (NP2/NPTX2), also known as neuronal activity-regulated pentraxin (Narp), is an immediate-early gene that has been shown to play a critical role in guiding synaptic plasticity. NP2 has been previously linked to excitatory neurotransmission, based on its ability to aggregate excitatory receptors in the central nervous system. The mechanisms mediating the effects of NP2 on excitatory neurotransmission remain unclear and warrants further investigation. This review article focuses on the biological features of NP2 and discusses the literature supporting a role for NP2 and other pentraxins in glutamatergic signaling. An analysis of evidence around the role of pentraxins in neuropathology is also reviewed.

## The Pentraxin Family

The pentraxins are a family of phylogenetically conserved molecules characterized by a cyclic multimeric structure. They are identified by the “pentraxin signature” sequence, His-x-Cys-x-Ser/Thy-Trp-x-Ser/Thy, where x is any amino acid (Breviarios et al., 1992). This family of proteins is divided into two major subfamilies based on their structure: short and long pentraxins. The short pentraxins include the C-reactive protein (CRP) also known as pentraxin 1 (PTX1; Whitehead et al., 1990) and the serum amyloid P component (SAP) also known as pentraxin 2 (PTX2; Dowton and McGrew, 1990), which are involved in regulating the innate immune response and are primarily produced in the liver. The long pentraxins include the neuronal pentraxins and pentraxin 3 (Breviarios et al., 1992; Lee et al., 1993). The neuronal pentraxins include neuronal pentraxin 1 (NP1, NPTX1; Schlimgen et al., 1995), neuronal pentraxin 2 (NP2, NPTX2) also known as neuronal activity-regulated pentraxin (Narp; Hsu and Perin, 1995; Tsui et al., 1996), and neuronal pentraxin receptor (NPTXR, NPR; Dodds et al., 1997). There are varying degrees of homology among the pentraxins (Table 1).

**Table 1:** Relative amino acid homologies among the pentraxin family.

Protein variant	NP1	NP2	Narp
CRP	20–30%^1^	Not known	24%^3^
SAP	20–30%^1^	Not known	26%^3^
Apexin	Not known	88%^1^	Not known
NPR	49%^2^	48%^2^	Not known
NP1	-	54%^1^	45%^3^
NP2	54%^1^	-	94%^3^

*^1^Hsu and Perin (1995), ^2^Dodds et al. (1997) and ^3^Tsui et al. (1996)*.

NP1 and NP2 are secreted glycoproteins (Schlimgen et al., 1995; Tsui et al., 1996), whereas NPR is a transmembrane protein (Dodds et al., 1997). The neuronal pentraxins have been implicated in α-amino-3-hydroxy-5-methylisoxazole-4-propionic acid (AMPA)-mediated excitatory synapse assembly (Lee et al., 2017). NP1 is suggested to aid in the synaptic reuptake of debris into the pre-synaptic cell or neighboring glial cells. NP1 and NP2 exist as multimeric complexes (Xu et al., 2003; Mariga et al., 2015). Although the exact function of this multimerization is unclear, the relative ratio of the components in the complex is dynamically dependent on the developmental stage and neuronal activity (Xu et al., 2003).

## Biochemistry and Structure of NP2

Like the other pentraxin molecules, NP2 is evolutionary-conserved and has been identified in several species. There are varying degrees of homology among the different protein variants (Table 2). The human homolog of NP2 was identified by Hsu and Perin (1995). They identified the protein as NPTX2 and the gene as *NPTX2*. The NPTX2 protein shares a 54% amino acid sequence homology with NP1 (Hsu and Perin, 1995). Tsui et al. (1996) identified the rat homolog of NP2 and termed the gene, *Narp*, and the protein as “Narp,” which was also characterized as an immediate early gene (IEG).

**Table 2:** Relative amino acid homologies of NPTX2 variants with the human protein form.

Animal	Gene	Protein	Homology with NPTX2	Chromosome location
Human	*NPTX2*	NPTX2/NPII	-	7q21.3-q22.1
Mouse/Rat	*Narp*	NP2/Narp	94%	Not known
Guinea Pig	Not known	Apexin	90%	Not known

While the precise structure of NP2 is not yet known, key molecular groups have been identified. NP2 contains both an N-terminal and C-terminal domain, and shares 69% homology with rat NP1 over its carboxyl end, and 90% overall homology with apexin, the guinea pig variant of the protein, most notably over its C-terminal domain (Reid and Blobel, 1994; Tsui et al., 1996). Furthermore, NP2 is a calcium-dependent lectin and is thus able to bind to ligands in a calcium-dependent manner (Schwalbe et al., 1992; Hsu and Perin, 1995). Finally, NP2 and its variants can form a cyclic pentamer structure (Hsu and Perin, 1995).

## Expression Pattern of NP2 in The Nervous System

Rodent NP2 mRNA is selectively expressed in neurons of the developing and adult brain and spinal cord (Tsui et al., 1996). Furthermore, *in situ hybridization* has revealed NP2 mRNA is expressed in the retinal ganglion cell (RGC) layer—specifically in RGC axons—and inner nuclear layer of the retina, where it shows both developmental and spatial variation (Bjartmar et al., 2006). NP2 is also widely expressed in the brain; with several reports showing its expression in the hippocampus, the dentate gyrus, cerebellum, cerebral cortex as well as in the medial habenula and the habenulo-interpeduncular pathway (Tsui et al., 1996; Reti et al., 2002). Unlike the other pentraxins, NP2 is not localized to a particular bodily system and its mRNA has been identified in the liver, kidney and the testes (Hsu and Perin, 1995; Tsui et al., 1996).

## NP2 and Ampa Receptors

AMPA receptors are one of the key glutamate receptors in the central nervous system and their dynamic trafficking is linked to various essential processes such as synaptic plasticity. Their trafficking, often *via* PDZ-domain-containing proteins, is essential for both long-term potentiation and long-term depression (Wang et al., 2005). NP2 has been consistently associated with AMPA-mediated excitatory synaptogenesis. O’Brien et al. (1999) found that 90% of GluR1 clusters on aspiny neurons in rat postnatal hippocampal cultures are associated with NP2, but *not* on synapses that are GAD-positive, suggesting that NP2 colocalizes preferentially with GluR1 at excitatory synapses. Additionally, NP2 induces clustering of GluR1, GluR2, and GluR3 in transfected HEK293 cells, suggesting a key role in AMPA-mediated excitatory synaptogenesis. A follow-up study found that impeding endogenous NP2 binding to the synaptic membrane significantly decreased GluR1 and GluR2 clustering in HEK 293 cell cultures (O’Brien et al., 2002).

**Figure 1:**
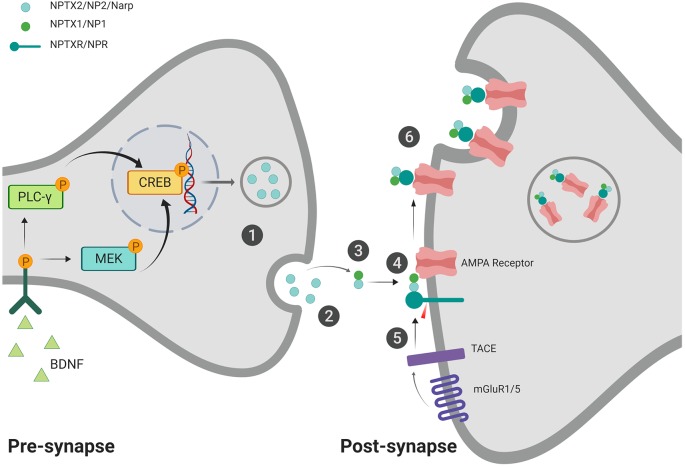
Proposed mechanism for neuronal pentraxin 2 (NP2)-facilitated α-amino-3-hydroxy-5-methylisoxazole-4-propionic acid (AMPA) receptor aggregation. (1) Pentameric NP2 is released from the pre-synaptic cell in response to neuronal activity/seizure (Tsui et al., 1996) or brain-derived neurotrophic factor (BDNF) induction (Mariga et al., 2015). (2) NP2 makes its way to the post-synaptic membrane, possibly facilitated *via* perineuronal nets (van’t Spijker and Kwok, 2017). NP2 may be maintained at the membrane through PNN interaction (not shown; Chang et al., 2010). (3) NP2 can form a complex with NP1 at the post-synaptic membrane (Dodds et al., 1997; Xu et al., 2003). (4) NP2 complex can aggregate AMPA receptors (O’Brien et al., 1999, 2002). The complex can then also associate with neuronal pentraxin receptor (NPR), which is anchored to the membrane through its transmembrane domain (Dodds et al., 1997; Cho et al., 2008). (5) In the presence of activated-mGluR1/5, Tumor Necrosis Factor-alpha Converting Enzyme (TACE) cleaves the transmembrane domain of NPR (Cho et al., 2008). (6) The cleavage of the transmembrane domain allows for the internalization of the neuronal pentraxin complex and associated AMPA receptors *via* endocytosis (Cho et al., 2008). This internalization process may represent a protective mechanism against excitotoxicity (Schwarz et al., 2002). Created with BioRender.com.

Recent work conducted by Lee et al. (2017) examined the interaction between NPR, NP1, and NP2 at the synapse, suggesting that NPR is a synaptic organizer which shepherds the maturation of excitatory synapses through interaction with AMPA receptors. They found that other NP’s (NP1 and NP2) could also play a critical role in stabilizing the complex through multimeric binding. NPR also directly interacts with GluA1 through its pentraxin domain (Lee et al., 2017). In HEK293 cells, NPR knock-out resulted in decreased expression of NP2 by 40%, and NPR overexpression increased NP2 levels by 2-5x, suggesting a relationship between their expression patterns. The NPR KO’s showed a decrease in excitatory post-synaptic specializations and impacted the levels of NP2 and NP1 at the synapse, suggesting it’s involved in stabilizing the pentraxins on the membrane (Lee et al., 2017). Furthermore, NPR, along with NP1, has been found to colocalize with GluR4 puncta in hippocampal neuron cultures, indicating an important role in functional synaptic recruitment of GluR4. Sia et al. (2007) propose that NPR binds to NP1, which can then bind to GluR4 and toggle it to the membrane (Figure 1).

In hippocampal neuron-glia co-cultures, NP2 was found at 50% of GFP-GluR4 puncta at neuron-glia excitatory synapses, and the colocalization decreased as the cultures matured. This supports the notion that NP2 is recruited during early synapse development. However, NP1 and NPR colocalized consistently with these puncta, regardless of the stage of synaptic maturity (Sia et al., 2007). This research is particularly important given that NP2 is often found colocalized with both NP1 and NPR in hippocampus and neocortex. Interestingly, NP2 is found to colocalize more with GluR2 in COS cell cultures than NP1 does, suggesting a possible role in aggregation of GluR2. Alone, NP1 has some capability to aggregate GluR2. However, the most effective aggregation seems to be when NP2 and NP1 are expressed together (Xu et al., 2003).

## A Proposed Mechanism for NP2 in Neuroplasticity and Neuroprotection

As an IEG, NP2 is upregulated in response to synaptic activity (Tsui et al., 1996). Mariga et al. (2015) found that NP2 has a bidirectional relationship with brain-derived neurotrophic factor (BDNF), wherein BDNF is able to induce NP2 expression even in the absence of neuronal activity. Once NP2 is released into the synapse, it can accumulate on the pre- or post-synaptic membrane surface with the help of perineuronal nets (PNN’s) *via* a calcium-dependent binding (Chang et al., 2010; van’t Spijker and Kwok, 2017). NP2 is unable to accumulate on the cell surface of parvalbumin interneurons if perineuronal nets are absent.

The function of NP2 on the membrane surface has not been fully elucidated. Possible mechanisms may involve NP2-mediated aggregation of AMPA receptors at the membrane (O’Brien et al., 1999, 2002). NP2 is able to form complexes with NP1, and together they can effectively aggregate GluR2 AMPA receptor subunits (Xu et al., 2003), suggesting a possible mechanism underlying NP2-dependent AMPA receptor aggregation and synaptic maturation.

Using HEK293 cell cultures, Cho et al. (2008) determined that upregulation of Tumor Necrosis Factor-α Converting Enzyme (TACE) can cleave the N-terminal transmembrane domain of NPR, releasing it from the membrane, thereby allowing it to cluster with NP2 and AMPA (Cummings et al., 2017). The cell can then internalize the complex through endosomal engulfment (Cho et al., 2008; Figure 1). TACE is upregulated in response to various environmental conditions, such as ischemic preconditioning (IPC) *in vitro* and *in vivo* (Cárdenas et al., 2002; Romera et al., 2004). This working theory may explain the recruitment and removal of AMPA receptors from the synaptic membrane and is in line with Schwarz’s prediction that the upregulation of NP2 protects neurons from glutamate excitotoxicity through internalization of AMPA receptors (Schwarz et al., 2002) and thus plays a role in neuroplasticity and neuroprotection.

## The Role of NP2 in Neurodegenerative Diseases

Fluctuating levels of NP2 has been identified in various neurological diseases. Previous work suggests that NP2 may be involved in regulating specific aspects of several neurological conditions. Herein, we focus our discussion on a role for NP2 in four conditions: epilepsy, Parkinson’s disease (PD), Ischemia, and Alzheimer’s disease (AD).

### Epilepsy

Aside from the initial discovery that NP2 mRNA is upregulated after maximal electroconvulsive seizure (MECS; Tsui et al., 1996), there has been very little research linking neuronal pentraxins and seizure. Tsui et al. (1996) found that NP2 mRNA was upregulated in the hippocampus as early as 1 h after seizure in Sprague–Dawley rats, remaining elevated for up to 8 h post-seizure in granule cell neurons, and rat cerebral cortex. Similar experiments revealed differential expression of NP2 and NP1 in the cerebellum and hippocampus after MECs (Xu et al., 2003). NP1 was elevated four times in the hippocampus and 50 times in the cerebellum after MECs. Changes in the distribution and diversity of GluR’s are a hallmark of synaptic plasticity and the progression of epilepsy (Bonansco and Fuenzalida, 2016), so it is possible that NP2, given its upregulation in MECS, may be involved in establishing these long-term AMPA-dependent changes at the synapse in epilepsy.

### Parkinson’s Disease

The human NP2 gene is overexpressed in the striatum after treatment with L-DOPA (Charbonnier-Beaupel et al., 2015). This treatment, if used long-term, has been shown to cause L-DOPA-induced dyskinesia (LID). NP2 KO mice show less severe LID after a single treatment, suggesting a role for the ERK pathway (Charbonnier-Beaupel et al., 2015). Human NP2 mRNA is also upregulated in sporadic forms of PD. Most of the NP2 identified in PD is found in Lewy bodies (Moran et al., 2008). Specifically, NP2 mRNA was found in cortical nerve cells as well as glial cells in the substantia nigra. Indeed, Moran et al. (2008) found that NP2 was the most upregulated gene in their genome expression profile. In their study, approximately one-third of the Lewy bodies also co-expressed NP2. Given that NP2 is upregulated in ceramide-dependent apoptosis (Decraene et al., 2002), NP2 may contribute to cell death in the substantia nigra (through AMPA receptor-mediated mechanisms) and the formation of Lewy bodies (Moran et al., 2008). Further research into the role of pentraxins in PD pathology is warranted.

### Ischemia

The role of NP2 in stroke pathology has been previously explored, particularly in the context of ischemic injury. The rodent *NP2* gene is mildly upregulated in the ipsilateral hemisphere of Sprague–Dawley rats after 30-min middle cerebral artery occlusion (MCAO). These data are aligned with the results from MECs (Tsui et al., 1996). The researchers speculate that NP2 upregulation may combat the excitotoxic glutamate release by prompting AMPA receptor internalization, though further work is required to validate this possibility (Schwarz et al., 2002).

Alternatively, NP2 may be involved in supporting GluR2-lacking AMPA receptors at the synapse. GluR2 subunits are unique in that they render an AMPA receptor impermeable to calcium. Transient forebrain ischemia causes a decrease in GluR2 mRNA (Soundarapandian et al., 2005) and an increase in GluR2-lacking AMPA receptors post-ischemia in CA1 neurons in the hippocampus (Noh et al., 2005). Oxygen-glucose deprivation (OGD) also triggers an increase in GluR2 endocytosis and increased presence of GluR1 and GluR3 at post-synaptic densities (Liu et al., 2006). This increase in calcium-permeable AMPA receptors likely contributes to neuronal cell death (Noh et al., 2005). An increase in GluR2-lacking AMPA receptors to the cell surface may exacerbate the Ca^2+^-dependent toxicity that is a well-known precursor to neuronal death. Perhaps, increased NP2 could be deleterious to neurons within the brain following ischemic insult while calcium levels are still elevated.

### Alzheimer’s Disease

AD is a progressive neurodegenerative disorder that is characterized, among other things, by the presence of amyloid plaques. Xiao et al. (2017) found that *nptx2*^−/−^rats with amyloidosis showed a decrease in GluR4 expression on parvalbumin interneurons compared to controls (Xiao et al., 2017). Human studies found decreased NP2 in human AD brains compared to brains that show amyloidosis but no cognitive impairment. In fact, there is a significant correlation between the level of human NP2 in CSF and the cognitive performance and hippocampal volume of patients with AD (Hanson, 2017; Xiao et al., 2017).

Parvalbumin interneurons are particularly susceptible to damage in the AD brain and are decreased in numbers (up to 60%) in the Alzheimer’s pathology compared to controls (Brady and Mufson, 1997). Parvalbumin interneurons selectively express GluR1 and GluR4 AMPA receptor subunits over other subunit types (Chen et al., 1996). The available literature suggests that the main function of NP2 could be to regulate specific AMPA receptor subunits in the parvalbumin interneurons.

Long neuronal pentraxin expression in AD is indicative of pentraxin-mediated AMPA regulation. NP1 and its fragments are upregulated in the brain and plasma of 7–8-month-old E4FAD mice (a transgenic mouse model of familial AD using APOE4 genotype), in 4 month-old WT mice with induced αβ burden, and in the plasma of patients with mild cognitive impairment (Ma et al., 2018). Interestingly, NPR is upregulated in soluble cortical homogenate fractions of 3–9-month-old APP/PS1 rats, but not at 18–20 months (Bilousova et al., 2015). The suggestion is that the lack of soluble NPR in the late-stage animals was due to a degradation in TACE activity (Qian et al., 2016) leading to less cleaved NPR in the soluble preparations. The increase in glutamate release that is evident before plaque deposition in AD mouse models (Cummings et al., 2015) may be due, at least in part, to the upregulation of NP1 and NPR (Cummings et al., 2017). NP2 is also downregulated in AD (Hanson, 2017; Xiao et al., 2017). Which may allow NP1 to exhibit its proposed deleterious effects at the synapse (Figueiro-Silva et al., 2015).

TACE (or ADAM17), the cleavage enzyme of NPR, is also able to act as an α-secretase for APP, resulting in a proposed neuroprotective by-product (Postina, 2012). TACE is also upregulated in AD (Skovronsky et al., 2001) but becomes inactive in the later stages of AD as the αβ burden increases (Bilousova et al., 2015). It could be that TACE is upregulated to combat the growing concentration of APP or in response to the increase in APP (Lüscher and Huber, 2010). An increase in active TACE would mean an increase in NPR cleavage, which is essential in the proposed mechanism for mGluR1/5-dependent LTD (Cho et al., 2008). However, while the increase in TACE and LTD may be a compensatory method of coping with the increased αβ burden, it also increases the presence of pro-inflammatory factors as TACE continues to cleave other molecules (Qian et al., 2016) exacerbating neuronal damage. Overall, the increase in NP2 and NP1, the increase of membrane-bound NPR, and the inactivation of TACE suggests improper AMPA trafficking (Baglietto-Vargas et al., 2018) that relies on pentraxin-dependent mechanisms.

## Concluding Remarks and Future Perspectives

A crucial next step in our understanding of NP2 in the nervous system is to define and characterize its role in AMPA receptor aggregation and internalization. NP2 has been linked to essential processes such as AMPA trafficking (O’Brien et al., 1999, 2002) and mGluR1/5-dependent LTD (Cho et al., 2008). These processes are pivotal in synaptic plasticity and excitatory synaptogenesis. However, there are many unanswered questions around the impact of NP2 on these processes, as is seen in the disease research. NP2 seems unequivocally involved in these pathologies, but we do not yet know, how, or to what extent.

While the mechanism proposed herein has its foundation in the research, there are many gaps that need to be addressed. We know that NP2 is upregulated in response to synaptic activity and BDNF (Tsui et al., 1996; Mariga et al., 2015), but we do not know what other downstream effects may be occurring. Furthermore, almost all research concerning NP2 has been conducted on neurons. It may be advantageous to turn our eyes to other cell types in the central nervous system to determine whether NP2 effects their function at the synapse.

The pathology research supports the proposed mechanism and links NP2 expression to disease progression. Indeed, many neuronal pentraxins are being considered potential biomarkers for neurological disease (Yin et al., 2009; Bilousova et al., 2015; Xiao et al., 2017). Several of the diseases discussed herein have characteristic calcium and glutamate increases. Given the role of NP2 in GluR binding and clustering, the link between cell survival and NP2 expression should be investigated. Establishing this will open new opportunities in therapeutic research.

In summary, NP2 is a long pentraxin protein linked to excitatory synaptogenesis, AMPA receptor aggregation and internalization, and various neurological pathologies. However, despite promising findings that suggest a role in neuroplasticity and excitotoxic protection, further research is warranted to more clearly characterize the mechanisms underlying these processes and define its implications for excitatory neurotransmission and excitotoxicity in the nervous system.

## Author Contributions

GC and US contributed equally to the manuscript. GC and US wrote the first draft of the manuscript. PS wrote sections of the manuscript. All authors contributed to manuscript revisions, read and approved the submitted version.

## Conflict of Interest

The authors declare that the research was conducted in the absence of any commercial or financial relationships that could be construed as a potential conflict of interest.
